# Challenges faced by teachers of postgraduate health professions blended learning programs: a qualitative analysis

**DOI:** 10.1186/s12909-024-05213-8

**Published:** 2024-03-07

**Authors:** Sana Shah, Usman Mahboob, Syed Muhammad Junaid, Sana Siddiqui, Brekhna Jamil, Saadia Rehman

**Affiliations:** 1 Islam Medical College, Sialkot, Pakistan; 2 Khyber Medical University, Peshawar, Pakistan; 3 Sardar Begum Dental College, Peshawar, Pakistan; 4 National University of Medical Sciences, Rawalpindi, Pakistan; 5 Abbottabad International Medical Institute, Abbottabad, Pakistan

**Keywords:** Blended learning, Online learning distance education, Faculty, Health occupations education, Educational technology

## Abstract

**Introduction:**

Blended learning, which integrates classroom face-to-face teaching with both asynchronous and synchronous online learning elements, has swiftly gained acceptance in educational environments. However, the implementation of blended learning presents challenges that impact all stakeholders, necessitating thoughtful consideration. Teachers play a central role in shaping the instructional experience among these stakeholders. To fully realize the potential of comprehensive blended learning, it is imperative to identify the challenges faced by these teachers and develop strategies that sensibly address and overcome them.

**Methodology:**

A qualitative exploratory study was conducted with twelve teachers involved in the postgraduate blended learning health professions program at Khyber Medical University, Peshawar in 2022. One-on-one semi-structured Interviews were conducted via WhatsApp/ZOOM, transcribed by Otter AI, coded on NVivo & analyzed using Braun/Clarke’s Thematic Analysis.

**Results:**

Three themes of challenges faced by teachers of postgraduate blended learning programs were constructed namely (1) Skills, such as (i) digital, (ii) instructional, and (iii) online class management, and (2) Administrative barriers in terms of (iv) resources (iv) training, and (3) Faculty resistance.

**Conclusion:**

This study provides profound insights into the daunting challenges that postgraduate blended learning program teachers encounter in terms of skills, administrative barriers, and faculty resistance. These findings offer a valuable opportunity for program directors to identify the critical requirements of these faculties in their pursuit of effective teaching and learning, ultimately transforming the landscape of blended education. This study emphasizes the need for ongoing faculty development and institutional support to address the identified challenges and improve the quality of postgraduate blended-learning programs.

## Introduction

Blended learning combines face-to-face and online learning activities into a single, integrated instructional approach, utilizing technology to deliver instruction, and learning materials beyond in-person sessions [1, 2]. This approach aims to retain some level of in-person interaction and engagement while reaping the benefits of both face-to-face and online learning modes (synchronous and asynchronous), including flexibility, pedagogical richness, and cost-effectiveness [3] The online component of blended learning refers to utilizing technology and the internet to deliver instruction and learning materials outside of face-to-face sessions, encompassing activities such as online discussions, virtual lectures, and other forms of digital communication and collaboration [4]. Blended learning can foster effective learning among postgraduate health care students [3]. Liu et al. conducted a comparative analysis, assessing the effectiveness of blended learning in health professions against traditional learning and fully online education. Their cautious conclusion suggests that blended learning more effectively facilitates knowledge acquisition when compared to both face-to-face and fully online education [5]. Additionally, consistent evidence across studies demonstrates superior knowledge outcomes with blended learning as opposed to traditional learning in health education which refers to the use of teaching and instructional strategies typically found in a teacher-led classroom, including didactic, drill, and practice [6].

Blended learning, while beneficial, poses challenges that hinder efficient teaching and learning such as insufficient institutional support, lack of direction, inadequate educator skills, and time constraints [2, 7]. The literature highlights challenges in creating Blended Learning Programs (BLP), such as incorporating flexibility, encouraging interaction, facilitating student learning, and fostering a positive learning environment [8].

Most of the academic study on challenges in blended learning is related to students’ perspectives such as retention, motivation, and engagement, among other things [9]. There is currently only a handful of literature that examines the evidence from the health educator's lens. The study will help explore the barriers and challenges faced by teachers who play a myriad of roles in blended learning health professions' postgraduate programs. The identification of these challenges will help devise strategies/recommendations that can help construct a foundation for faculty development and training on an ongoing basis, as well as for the overall enhancement of the quality of the postgraduate health professions programs.

## Methods

### Study design & setting

This qualitative exploratory study was conducted at the Post Graduate Health Professions Education Institutes of Khyber Medical University in Peshawar, Khyber Pakhtunkhwa, Pakistan. The study focused on constituent colleges and institutions of the university that offered blended learning (BL) programmes at the postgraduate level.

### Study participants

The purposive sampling method was employed to select participants, and the study aimed to explore the challenges of teachers who were engaged in both online (synchronous, asynchronous) and onsite, face-to-face teaching components of BL programmes. The study excluded teachers not engaged in either component of the BL programme and those who were unwilling to participate. A possible definition of a teacher in health education is an educator who possesses strong, professional competencies in health education, including a broad range of knowledge, pedagogical skills, and attitudes that are conducive to the promotion of health. Such a teacher is capable, competent, and skilled in providing health education to future adults, and is uniquely positioned to contribute to a nation's health gain through the provision of health education to students [10].

### Research instrument

To enhance the trustworthiness of the interview guide within a constructionist paradigm inspired by the Community of Inquiry framework was developed. The questions underwent a rigorous pilot refinement process. Triangulation was employed, involving expert opinions from seasoned medical educationists and research faculty at the under-studied university, following the guidelines outlined in AMEE guide no. 87. Additionally, a constructive approach was taken with cognitive interviews, during which the researcher engaged in discussions with colleagues from the public health and medical education specialty. The interview guide itself was piloted, incorporating feedback from a small group of participants. This iterative process, guided by both expert input and constructive dialogues, aimed to enhance the relevance, clarity, and comprehensibility of the interview guide, fostering a more nuanced exploration of participants' experiences within the Community of Inquiry framework. This framework aims to capture the educational dynamic and guide research on the efficacy of online learning in higher education. The initial presumption was that a learning experience aimed at achieving higher-order learning goals functions best when it is integrated into a community of inquiry made up of both students and teachers to Lipman's (2003) argument, a community of inquiry is essential for both the operationalization of critical or reflective thinking and as a teaching strategy. This presumption was in line with Dewey's (1959) educational philosophy, which defined education as the collaborative reconstruction of experience. This study was conducted in a community of inquiry within the context of a collaborative, constructivist learning experience. In a community of inquiry, there is both freedom and collaboration (co-regulation). The interview guide, inspired by the Community of Inquiry Framework consisted of the following questions:

i. What does a blended learning programme mean to you?
ii. What is your experience of a blended learning programme?
iii. What were the barriers to establishing communication & providing a supportive environment?
iv. What were the challenges related to the delivery of feedback?
v. What were the challenges that you faced in the course construct/design of health professions’ blended learning programs?
vi. What were the challenges in designing course learning activities?
vii. ‘What were the challenges in implementing activities to encourage engagement between and among students?
viii. What were the challenges in implementing activities to encourage engagement between the teacher and the student?
ix. What were the challenges you faced regarding the assessment and certification of student learning?
x. Any more challenges that you would like to discuss?

### Data collection

Semi-structured interviews were conducted one-on-one with the principal researcher and were audio-recorded with the participant's consent. Participants were given an information sheet and the interview questions beforehand. Date and time of the interview were scheduled at the participant’s convenience. The interviews were conducted through WhatsApp or Zoom Meetings. The participants were asked, and the transcripts were generated using Otter AI software, which allowed for real-time transcription, editing, and exporting of the transcripts as text.

### Data analysis

The data analysis adhered to Braun and Clarke's thematic analysis, involving an iterative process with six steps. In our collaborative approach, SS conducted the initial coding, and UM provided a countercheck on the codes. This involved close supervision and refinement of codes through joint discussions to ensure a robust and comprehensive coding framework. Both researchers engaged in ongoing discussions to develop themes, striving for group agreement throughout the analysis process. Initially, we employed inductive coding to let emerging patterns shape the coding structure. Subsequently, we incorporated deductive coding, aligning codes with theoretical perspectives, particularly guided by the Community of Inquiry (CoI) framework.

The Community of Inquiry theory served as a foundational framework for the deductive coding process, offering a structured lens to interpret and categorize the data. This theoretical perspective refined and deepened our analysis, providing coherence to the emerging themes. In the stages of thematic analysis, coding was the primary step. We identified words and phrases with conceptual similarities, translating them into codes. These codes were then organized into groups, forming the basis for subsequent theming. This process involved grouping codes to create overarching concepts, further categorized into themes and subthemes.

With a sample size of 12 participants, saturation was determined when subsequent interviews yielded no new insights or themes [11]. This point signified a comprehensive understanding of the subject matter, affirming that the data collection process had captured the diverse perspectives and nuanced details related to the challenges faced by teachers engaged in both online and onsite components of the blended learning program. The study was conducted with ethical considerations, and the anonymity and confidentiality of the participants were maintained throughout the study. The participants were informed of the study's purpose, and their voluntary participation was ensured. The study was approved by the Institutional Review Board of Khyber Medical University.

### Reflexivity

In line with a constructionist paradigm, both researchers (SS and UM) consistently acknowledged their perspectives and biases throughout the study. Regular discussions ensured transparency and a nuanced understanding of teachers' challenges in blended learning programs.

### Trustworthiness

In ensuring the trustworthiness of their study within a constructionist paradigm, the researchers implemented several key strategies. Firstly, they maintained prolonged engagement, immersing themselves extensively in the data and context over time, which facilitated a more profound and nuanced understanding. Second, member checking played a vital role as participants actively engaged with interview summaries, sharing insights, and confirming the alignment of the researchers' interpretations with their experiences. Third, external perspectives were incorporated through peer debriefing, involving discussions with non-involved colleagues, thus enhancing the credibility of the analysis. The fourth strategy involved credibility in coding, where collaborative efforts by SS and UM were seamlessly integrated to fortify the robustness of the coding structure. Lastly, reflexivity was actively woven into their approach, ensuring constant reflection on biases and perspectives to maintain the dependability and authenticity of their study.

## Results

The study involved 12 participants from diverse postgraduate medical education fields, engaging in programs like Medical Education, Public Health, Physiotherapy, and Basic Medical Sciences Table 1 shows the summary of participant characteristics as well as details of the blended programs they were teaching. A total of 153 codes were generated, leading to the delineation of three overarching themes—Skills, Administrative challenges, and Faculty resistance—along with their respective subtheme.

**Table 1:** Participant profile and blended program structure summary

Total Participants 12					
Average Age 28–50 years					
Gender 5 males, 7 Females					
**Area** of Expertise	**Blended Program Structure**	**No. of Teachers from Specialty**	**Group Avg. Years in Educational Practice**	**Total Duration of Program**	**Nature of Employment**
Medical Education	In-person sessions for real-time engagement	3	8 years	2 yrs	Permanent
	Virtual sessions on Zoom with both synchronous and asynchronous components				
	Weekly synchronous virtual sessions for discussions and lectures				
	Learning management system includes uploaded materials for self-directed learning. Asynchronous elements pre-recorded lectures and discussion forums				
Public Health	In-person sessions for real-time engagement	3	10 years	2 yrs	
	Virtual sessions on Zoom with both synchronous and asynchronous components				
	Weekly synchronous virtual sessions for discussions and lectures.				
	Learning management system includes uploaded materials for self-directed learning.				
	Asynchronous elements pre-recorded lectures and discussion forums				
Physiotherapy	In-person sessions for real-time engagement	3	5 years	2 yrs	
	Virtual sessions on Zoom with both synchronous and asynchronous components				
	Weekly synchronous virtual sessions for discussions and lectures				
	Learning management system includes uploaded materials for self-directed learning.				
	Asynchronous elements pre-recorded lectures and discussion forums				
Basic Medical Sciences	In-person sessions for real-time engagement	3	10 years	2 yrs	
	Virtual sessions on Zoom with both synchronous and asynchronous components				
	Weekly synchronous virtual sessions for discussions and lectures				
	Learning management system includes uploaded materials for self-directed learning.				
	Asynchronous elements pre-recorded lectures and discussion forums				

The thematic analysis illuminated challenges through the lens of the Community of Inquiry Framework (COI). Three overarching themes—Skills, Administrative challenges, and Faculty resistance—were constructed, each with distinctive subthemes, providing a nuanced understanding of the complexities faced by educators in the context of postgraduate blended learning program.

The Skills challenge highlighted the necessity for updated technological knowledge. Participants expressed difficulties in adapting to new technologies, such as applications for face-to-face sessions. They also noted challenges in email communication and the transition to online teaching, emphasizing the need for continuous skill development. For instance, a medical educationist stated, "One of the challenges can be you have to get updated with the technologies…new apps coming up like Mentimeter, which you can utilize during face-to-face sessions".

Administrative Challenges encompassed issues like internet connectivity, resource availability, and the need for skill training. Participants faced disruptions in communication due to internet issues and emphasized the importance of skill training for effective course design. A Professor of Public Health expressed, "Let's be honest, my communication was disturbed because most of the time, although the internet was not available".

Faculty Resistance emerged as a significant challenge, with participants expressing apprehension and struggles in transitioning to online teaching. The new digital medium posed difficulties, and faculty members found it challenging to engage effectively. One participant from the basic sciences faculty highlighted, "Faculty apprehension hampers communication…consider it a major issue".

In summary, the challenges identified in this study reflect the intricate nature of transitioning to blended learning. The participants' experiences, captured in the representative quotes, offer valuable insights into the multifaceted aspects of these challenges, shedding light on the complexities faced by educators in postgraduate blended learning programs (see Table 2 for a detailed presentation of quotes).

**Table 2:** Challenges identified through the lens of community inquiry framework (coi) including challenges in social presence, teaching presence and cognitive presence

Theoretical Framework Social Presence						
**Theme**				**Subtheme**		**Representative Quotes**
1		**Skills**		Digital		*“And one of the challenges can be you have to get updated with the technologies that are for one session, especially even for the face to face because there are new apps coming up like mentimeter *etc*., which you can utilize during face-to-face session to improve your social engagement with the audience”* *“So, the thing is, we are still very much used to paperwork and file, so I'll just start with simple thing we don't look at the emails, a lot of people a lot of faculty and students they miss their emails, the don’t know what spam folder is, what junk folders and email is even”*
				Instructional		*“So, people think teaching online easy, let me tell you it’s hard esp. designing a course that fosters effective communication.”* *“Missing nonverbal, visual body language cues messed with my communication”*
				Online Class Management Time Interpersonal		*“I must give them individual feedback. So that takes more time for me because I have to keep on working even the next morning. I have to edit and upload detailed feedback. So that kind of increases my work.”* *“Giving timely feedback was a struggle for me ‘* *“I had a difficulty to gather all the 20 to 25 students during one time because they were facing the problems of getting communicated and then getting connected with the net.”* *“So, in online especially unmotivated students who resist to turn on using cameras, and you call them one by one then managing such students and communicating with them is a bit of a baggage.”* *“Sometimes they even fake that we can’t hear you clearly.”* *“Students pretended they couldn't listen to us all of a sudden when they didn't know the answers to the question.”* *“Because you can communicate with students *via* resources before entering to the class *via* emails, WhatsApp groups, and you can talk about them, and they can also ask questions and they can play the concepts. However, the only problem is what to do when they say we have not received it they have not seen their mails, they have not checked the WhatsApp groups, urgh frustrating!”* *“I was comfortable with smaller groups of 5–6 students but when the strength increased to 40–45, I struggled to establish communication with them and had to come up with strategies that demanded a lot of time and effort.”* *“You know I’m not from around here, and I had a diverse group of students in terms of ethnicity, cultures, I, unfortunately, it was hard for me to effectively manage and communicate with them”*
2		**Administrative**		Resources Training		*“But let's be honest my communication was disturbed because most of the time although the internet was not available, and as I said, I'm quite used to I always maintaining good internet connectivity at my home or wherever I travel, so at the institution, the internet was a problem.”* *“And secondly, for postgraduate education, as BLP facilitators, we need more skill training on evidence-based strategies to design and conduct BLP courses. I’d like to know more about how to engage my audience”*
3		**Faculty Resistance**		Apprehension		*“Faculty apprehension hampers communication, without a doubt, I consider it a major issue.”*
**Theoretical Framework** **Teaching Presence**						
**Theme**				**Subtheme**		**Representative Quotes**
1		**Skills**		Digital		*“The people who were naive to digital tools and teaching techniques, who are not very much used to technology and computing kept complaining.”* *“It was difficult for us to teach in online because of the new medium.”* *“I want to emphasize on the fact that for most of the faculty members, it was a transitional change of incorporating the online teaching and online assessment with the on-campus one, so they struggled a lot, and they were quite apprehensive as compared to somebody like me who's already familiar with these types.”* *“So BLP was in itself quite a challenge when we were asked to do so. Because definitely, it was something very new in a digital aspect I must say, we hadn't done this before”*
				Instructional		*“Constructing LO’s for BL program was hard, tough to design compared to face to face* "*When it comes to online classes, designing, teaching the content as well as engaging the students in actual learning, is quite challenging*.” *“I divided my course content into theoretical part and the practical part. So basically, could teach cognitive and the psychomotor domain, but the affective domains could not be touched, and it was hard for me to teach it.*” *“Deciding content of online video tailored to a blp needs, recording the lecture and then uploading it was difficult for me.”* *“No specific problems but engagement was hard”*
				Online Class Management Time Interpersonal		*I will say that I started feeling nausea every time I was teaching Bloom's Taxonomy, because I was teaching it like redundantly, straight long every week.”* *“I remember there were times when I used to be up all-night designing activities and providing feedback, God it was exhausting.”* *“So, you have to be creative in designing BLP course and for being creative. You need to think you need to have time and a lot of resources to be used.”* *“Online teaching is hard; I think that requires a lot of preparation.”* *“You know, when I want to engage them, I have to stop my thinking and thought processes, and then call them up, I want to see them for their facial expressions, but the cameras are off and their names are there, so it gets annoying, it’s like I’m talking to a box.”* *“You got a screen, you don't know who you've got, a name is there, and you don't know who's the person sitting behind there? Who wants to really ask the question and who has understood it? So, I didn't find BLP very effective, it killed my teaching vibe”*
2		**Administrative**		Resources Training		*“I think we need instructional designers in our Institute, if you want to do a proper online and blended learning systems in our universities and institutions.”* *“So, I think it requires support from the leadership when it comes to the design of course and learning activities that establish a good teaching presence. It requires a lot of resources, IT support personnel’ and it requires a lot of training of faculty as well.”* *“So, it is very important for the facilitator and for the learners, that they should be given some very good orientation regarding the online activities that you're going to deliver* *My faculty was not trained to conduct or design BLP”*
3		**Faculty Resistance**		Apprehension		*“If a faculty is not motivated or trained enough, any sort of blended learning or any kind of engagement just doesn't work at all”*
**Theoretical Framework Cognitive Presence**						
**Theme**			**Subtheme**		**Representative Quotes**	
1	**Skills**		Digital		*“I couldn’t assess true body language facial expressions, couldn’t judge whether they were able to construct meaning “”*	
			Instructional		*“The questions in the online discussions and forum were not of higher order cognitive thinking they were off lower order cognitive thinking. So, students could just simply, easily get the answers without reading or understanding what they're writing.”* *“Ensuring academic integrity and transparency was a challenge so we avoided all kinds of e-assessments.”* “*So, the challenges were that we were not able to evaluate their learning in online as good as we could during the on-campus class session”*	
			Online Class Management Time Interpersonal		*“We couldn’t handle time in the online discussions, it was cumbersome for the faculty* *Managing their(student) screen absence is a major challenge for me to understand whether the concept that I’ve been delivering has been grasped pr not.”* *“I failed to observe in larger strengths of students that everyone has contributed, or everyone has learned or if there is any uniformity in learning of each and every individual.”* *“One of the key issues is how to make them “focus on session, because student can get lost on the virtual session quite easily, as they can have their own work or they're sitting in front of a laptop. So, it can be difficult in that sense to assert whether the content is making sense to them or not.”* *“In the online session students seem that they sit back, and they're turn off cameras and don’t participate in any discussion. So, I feel that to bring things forward and clarify the concepts, discussion is vital”*	
2	**Administrative**		Resources Training		*“We faced issues in terms of absence of reflection-portfolios, video-based learning platforms, collaborative platforms, learning management systems, *etc*.”* *“Lack of proctoring apps where we could ensure academic integrity and transparency.”* *“To be honest, I’m not aware of any instructional strategies to enhance cognitive presence in online courses, like I guess could use some training on this”*	
3	**Faculty Resistance**		Apprehension		*“I’ve seen teachers who were skeptic of BLP before even giving it a try, they just don’t like any innovation. They never involved themselves in online discussions, forums or any digitally superior activity”*	

## Discussion

**Fig. 1:**
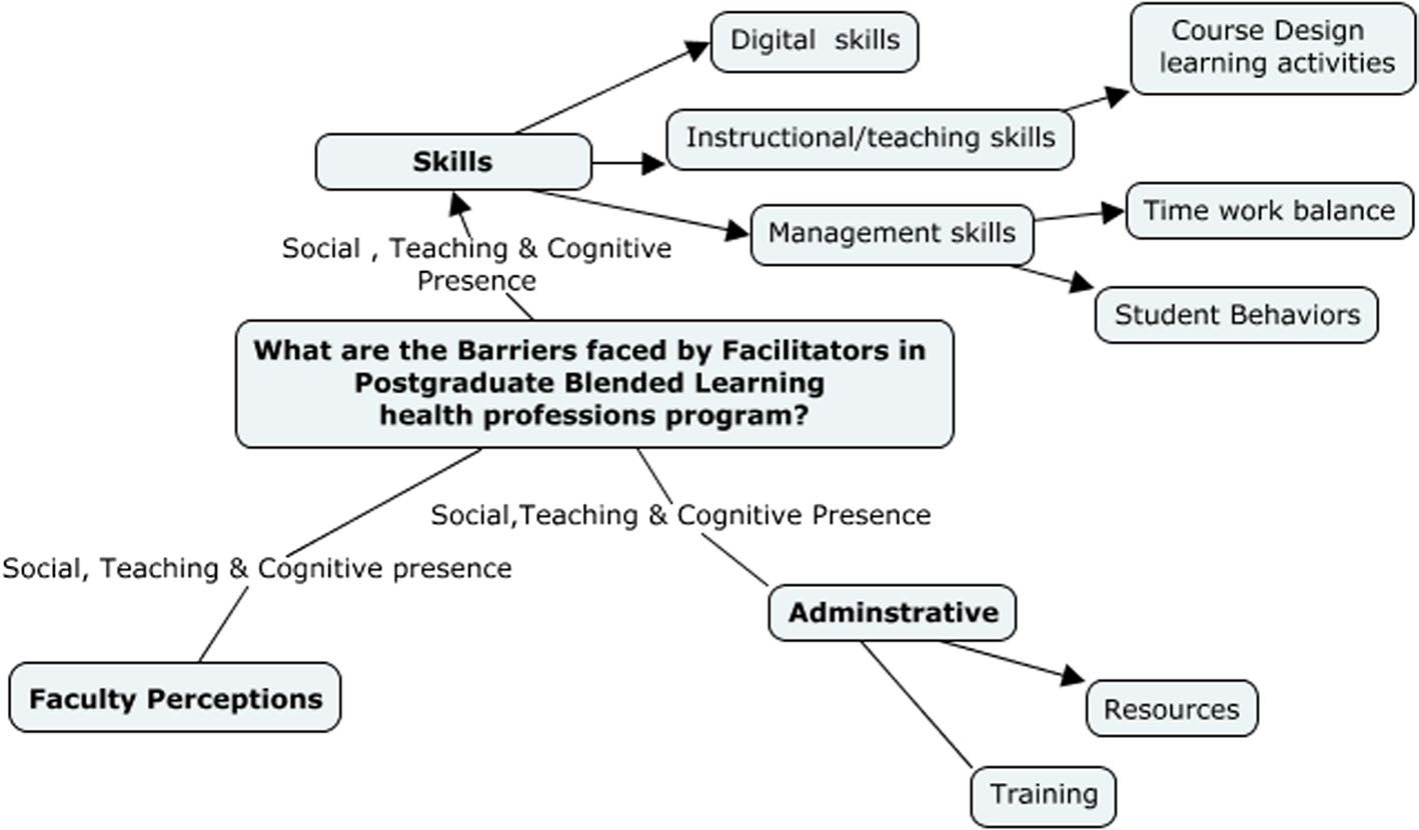
Concept map of the barriers faced by teachers in pg bl health professions program

The successful facilitation of postgraduate blended learning programs requires a comprehensive set of skills, knowledge, and resources. Our qualitative research study identified three key themes of challenges faced by teachers of these programs, including skills, administrative barriers, and faculty perceptions visualized through the lens of social, teaching, and cognitive presence in a blended learning program as shown in Fig. 1 as a concept map.

### Skills

Educators require competencies to adeptly navigate and manage diverse aspects of the learning experience. Insufficient skills posed a challenge for teachers, which was explored through the subthemes of Digital, Instructional, and Management skills.

#### Digital skills

One of the key challenges faced by teachers is acquiring and maintaining digital skills, as the integration of technology into blended learning necessitates technological expertise. This can be particularly daunting for educators who are not technologically savvy and can impact the quality of the learning experience for students. Given the prevalence of Gen Z students in our classrooms, competent with technology, the need for educators to possess digital skills has never been more critical. This may involve taking training courses on the use of digital tools and technologies, collaborating with colleagues who possess relevant skills, or engaging in self-directed learning to acquire the necessary knowledge and expertise. By doing so, educators can better cater to the needs of digital learners and provide them with a high-quality blended learning experience. As the researcher suggests, we are now in the age of the "survival of the digitalist," and educators must possess the skills and knowledge necessary to effectively facilitate blended learning programs to meet the needs of their students [12].

Studies reveal that adopting growing digital tools in blended learning is frequently problematic for academic staff [13].

#### Instructional skills

Conducting an effective blended learning program posed challenges in instructional skills. The online learning component of blended learning can be more cognitively challenging than in-person class discussions [14]. The absence of experience in developing pedagogical content for learning management systems is recognized as a challenge in the previous literature [12]. Teaching and designing a blended learning course necessitates a high level of expertise, as well as cognitive abilities that facilitate the intersection of social cognitive and teaching presences. Participants grappled with a key challenge: creating engaging content and activities due to a lack of evidence-based strategy knowledge. This struggle impacted teaching, social interactions, and cognitive presence. Assessment challenges, including transparency and tool selection, were also evident.

### Online class management skills

The term "online instructional/class management" refers to the vast range of abilities and strategies instructors employ to keep their pupils on track, focused, orderly, and academically productive throughout an online learning session [13]. Management skills, including practice, moderation, timekeeping, regulating student attitudes (motivation, focus engagement), and conscience to implement self-directed learning, are required to succeed in conducting blended learning courses.

When transitioning to online teaching, instructors must learn new time management skills as it requires time to learn new technology when teaching a blended course [15]. Literature suggests that online instructors are spending three times more time than face-to-face instructors evaluating student work [16]. Timely feedback suffered due to workload and online dynamics, causing delays from a lack of real-time interaction. Asynchronous discussions, while enhancing interactivity, presented time management difficulties for instructors.

In online environments, interpersonal interactions, considered crucial for learner-instructor engagement, foster a sense of community and connectedness, contributing to a strong bond with the course. Interpersonal contacts are valued highly by instructors and students alike [17]. Teachers also struggled to recognize when students are having difficulties and frequently fail to interact and communicate with them. Lack of familiarity with students, the absence of visual clues, the inability to regulate the strength and diversity of students, and, most importantly, ignorance of methods to regulate student behavior all seriously impede engagement, communication, and discussion about issues. As literature suggests teachers' enthusiasm is influenced by the attitudes of the students [18]. Balancing students' participation in both asynchronous and synchronous modes posed a challenge for teachers. The results of this study suggest a connection between challenges in guiding student interactions and issues such as a lack of motivation, focus, and engagement. These challenges had adverse effects on both the learners' social presence and cognitive engagement. This impact manifested in their interaction with others and their engagement with the content. Blended learning settings must promote a stimulating and productive learning atmosphere where students feel secure, appreciated, and welcomed while also encouraging favorable attitudes toward the subject matter and the teacher [19]. According to research, a supportive environment for learning can speed up the learning process and produce positive student outcomes including self-motivation, creativity, and wellbeing [20]. The term "managing student strength" in our study refers to the challenges associated with effectively handling and facilitating the learning experiences of a larger student cohort within the context of online education. It encompasses various aspects, including but not limited to maintaining active student engagement, fostering meaningful interactions, and ensuring optimal academic performance in a setting with an increased number of students. Teaching a larger online class posed difficulties, as teachers noticed reduced engagement among students (Social Presence) with each other and the instructor and less involvement with the course content (Cognitive Presence). Our findings match existing studies, indicating that larger groups are linked to lower individual student performance. Achieving effective collaborative learning, marked by coordinated actions in understanding and solving problems, becomes harder as the group size increases [21]. This emphasizes the need for careful approaches in managing a growing number of students in online education.

### Administrative

Institutional support is critical for providing opportunities for teachers to appreciate the significance of their role, as well as the role of technology, in the success of blended learning [22]. Our teachers' institutional support challenges can be broadly classified as resource and training-based.

#### Resources

Teachers must be provided with the required technical and educational resources by their institutions to be motivated to fully integrate technology with traditional face-to-face teaching in a proportional manner [23].

Learning through technology necessitates dependable hardware, simple software, and a high bandwidth network connection, in addition to skilled, trained personnel who support users and maintain the system.

According to our findings, the infrastructure lacked a reliable internet connection and a dedicated learning management system that supported integrating digital teaching tools such as video conferencing, e-portfolios, and e-assessments. The system lacked the necessary technical and human resources to support teaching, learning, and assessment.

Connectivity issues were identified as a significant impediment. Keeping in mind that as technological advances spread, internet bandwidth, dependable and accessible hardware, digital apps, and support should become less of an issue.

#### Training

This study identified a barrier as a lack of institutional support in terms of adequate training and faculty development programs to equip faculty with the necessary skills. The lack of awareness of evidence-based communication strategies was highlighted. Inadequate formal training in communication and feedback delivery strategies, as a result, compromises all three energies (social, teaching, and cognitive) required to ignite the promise of blended learning education.

Literature suggests that workshops should provide instructors with information on e-learning teaching models, approaches, tools, and frameworks [24]. A range of training sessions should be offered, including those on advanced tools, evaluation, content production, quality assurance, and blended learning. Also, accessibility should be just-in-time assistance. Throughout the programme, teachers struggled since they had not received this training. Failure of teachers to apply educational theories in course design and activity execution can also hurt the effectiveness of the blended learning programme.

### Faculty perceptions

The success or failure of a course is determined by attitudes, and as teachers are the main motivators, their attitude is crucial. Teaching in a blended learning setting is hampered by faculty understanding and a lack of desire. The main obstacle to the deployment of blended learning is the lack of faculty enthusiasm to include technology in their classes. According to studies, most professors do not believe that online teaching and learning are legitimate. According to research, the intrinsic drive of the faculty to teach is linked to accountability, increased student exchanges, and better learning outcomes [23]. Some teachers showed stronger intrinsic motivation and a preference for face-to-face courses due to improved satisfaction, urgency, and accountability. A sizeable portion of faculty members believe that face-to-face instruction is superior to online instruction, and they are more apprehensive than enthusiastic about instructing online [24]. As a result, there is a significant gap between students who want an online education, university administrators who want to see more students enroll, and faculty members who are reluctant to teach online.

Blended learning teachers expressed skepticism about the effectiveness of online instruction in improving learning. This doubt represents one of the observed negative opinions and beliefs held by teachers regarding the use of technology for teaching. The statistics show that instructors believed that online students learned less and that they needed more time to teach online. Additionally, faculty members stated that they believed that: (a) face-to-face interactions were more effective; (b) online interactions were less effective; (c) students took online courses because they believed them to be simpler; and (d) sensitive subjects shouldn't be taught online. Outstanding online educators, according to research, were challengers, affirmers, and influencers who created communities of inquiry and had a "strong social, cognitive, and teaching presence." The lack of a student-faculty link in asynchronous communication systems, according to the faculty, led to more time being spent on the course reading individual postings.

The current challenge in global online education is that administrators are highly enthusiastic about expanding online programs, whereas academics are more apprehensive than excited about this trend. Most faculty members are concerned about the expansion of online education, and they also think that the quality of online learning outcomes is lower. Our research also shows that faculty members shared similar viewpoints, considering it to be less effective than conventional teaching strategies and unfit for imparting information on health education. Overcoming this belief and ongoing opposition to teaching in a mixed-learning setting was a significant challenge.

### Considerations

This study pioneers an examination of barriers in postgraduate health programs through the Community of Inquiry Framework. Findings are practical for program improvement.

Interviews were conducted in English to align with academic discourse, broadening accessibility. However, this may have hindered participants' full expression due to language differences.

The absence of student and administrative perspectives limits the study. These factors should be considered in interpreting findings, emphasizing the need for future research improvements.

## Future implications

This study highlights the importance of continued faculty development and institutional assistance in addressing the problems faced by postgraduate blended learning programme teachers. The study makes the following suggestions: putting more emphasis on faculty development; developing a culture of support; removing administrative obstacles; enlarging the scope of research; and assessing programme effectiveness. Programme directors can improve the postgraduate blended learning programmes' quality and foster an environment that fosters efficient teaching and learning by putting these tips into practice.

More studies should be conducted to guide teachers and administrators on how to create successful blended learning programmes, and input from teachers, students, and administrators (all stakeholders) should be sought on how to address the barriers encountered in the implementation of blended learning programmes. As technological advances expand, new types of blends will develop, and education will be blended with various technologies but the basic question to be addressed will stay the same as technology advances spread: "How can such learning environments be organized to efficiently assist learning??"

## Conclusion

The study's findings give a thorough understanding of the difficulties postgraduate blended learning programme teachers face, including lack of skills, lack of administrative support, and faculty resistance. These challenges hinder the faculty from unlocking the full potential of a blended learning program. The findings give programme directors the chance to pinpoint essential conditions for efficient instruction and learning while also changing the face of blended learning. It is crucial to prioritize ongoing faculty development and institutional support to address the challenges identified in this study and improve the quality of postgraduate blended learning programs. Furthermore, program directors can use these findings to develop targeted interventions that address the specific needs of their faculty and enhance their teaching skills. This study underscores the importance of investing in the professional development of faculty to meet the evolving demands of blended education and improve student learning outcomes.

## Data Availability

Data is not shared publicly because permission for open public access has not been granted. it is safely stored on a server. But it is available from the corresponding author on reasonable request**.**

## Abbreviations

ASRB: Advanced Studies and Research Board
BL: Blended Learning
CoI: Community of Inquiry Framework
IHPER: Institute of Health Professions Education &Research
KMU: Khyber Medical University
LMS: Learning Management System
PG: Postgraduate
